# Multidrug-Resistant Coagulase-Negative Staphylococci Isolated from Bloodstream in the uMgungundlovu District of KwaZulu-Natal Province in South Africa: Emerging Pathogens

**DOI:** 10.3390/antibiotics10020198

**Published:** 2021-02-18

**Authors:** Jonathan Asante, Bakoena A. Hetsa, Daniel G. Amoako, Akebe Luther King Abia, Linda A. Bester, Sabiha Y. Essack

**Affiliations:** 1 Antimicrobial Research Unit, College of Health Sciences, University of KwaZulu-Natal, Durban 4000, South Africa; hetszab@gmail.com (B.A.H.); amoakodg@gmail.com (D.G.A.); lutherkinga@yahoo.fr (A.L.K.A.); essacks@ukzn.ac.za (S.Y.E.); 2 School of Laboratory Medicine and Medical Sciences, University of KwaZulu-Natal, Durban 4000, South Africa; 3 Centre for Respiratory Diseases and Meningitis, National Institute for Communicable Diseases, Johannesburg 2131, South Africa; 4 Biomedical Research Unit, University of KwaZulu-Natal, Durban 4000, South Africa; besterl@ukzn.ac.za

**Keywords:** coagulase-negative staphylococci, antibiotic resistance, multidrug resistance, infections, multiple antibiotic resistance index, public health

## Abstract

Coagulase-negative staphylococci (CoNS) are increasingly associated with nosocomial infections, especially among the immunocompromised and those with invasive medical devices, posing a significant concern. We report on clinical multidrug-resistant CoNS from the uMgungundlovu District, KwaZulu-Natal Province, South Africa, as emerging pathogens. One hundred and thirty presumptive CoNS were obtained from blood cultures. Culture, biochemical tests, and the Staphaurex™ Latex Agglutination Test were used for the initial identification of CoNS isolates; confirmation and speciation were undertaken by the VITEK 2 system. Susceptibilities of isolates against a panel of 20 antibiotics were determined using the Kirby-Bauer disk diffusion method, and the multiple antibiotic resistance (MAR) indices of the isolates were determined. The polymerase chain reaction (PCR) was used to amplify the *mec*A gene to confirm methicillin resistance. Overall, 89/130 presumptive CoNS isolates were confirmed as CoNS by the VITEK 2 system. Of these, 68 (76.4%) isolates were putatively methicillin-resistant by the phenotypic cefoxitin screen test and 63 (92.6%) were *mec*A positive. *Staphylococcus epidermidis* (19.1%), *S. hominis* ssp. *hominis* (15.7%), and *S. haemolyticus* (16.9%) were the most common CoNS species. Isolates showed high percentage resistance against penicillin (100.0%), erythromycin (74.2%), and azithromycin (74.2%) while displaying high susceptibilities to linezolid (95.5%), gentamicin (95.5%), and tigecycline (94.4%). Multidrug resistance (MDR) was observed in 76.4% of isolates. MAR index calculation revealed 71.9% of isolates with MAR index >0.2 and 20.2% >0.5. Isolates with the highest MAR indices (0.7 and 0.8) were recovered from the neonatal intensive care unit. Fifty-one MDR antibiograms were observed. The high prevalence of methicillin resistance and multidrug resistance in several species of CoNS necessitates surveillance of this emerging pathogen, currently considered a contaminant of microbial cultures.

## 1. Introduction

Staphylococci are classified as either coagulase-positive or coagulase-negative, depending on their ability to clot plasma that is facilitated by the enzyme coagulase [1]. Coagulase-negative staphylococci (CoNS) are the most frequent colonizers of the skin and mucous membranes and the most frequently isolated organisms in microbiology laboratories [2]. Although CoNS are mostly considered contaminants in clinical specimens, they have been implicated in clinically relevant infections, including urinary tract infections, endocarditis, bloodstream infections (including neonatal sepsis), and foreign body-related infections [2]. The skin and mucous membranes of the host, which are home to an abundance of CoNS species, are significant sources of endogenous CoNS infections, facilitated by transmission through medical procedures [3]. 

Pathogenic CoNS are usually associated with clinical environments and found in immunocompromised patients and patients with indwelling metallic or polymer devices, such as orthopedic prostheses, peripheral venous catheters, and artificial pacemakers; however, they are less commonly involved in community-associated diseases [3,4,5,6]. CoNS are the most common pathogens implicated in nosocomial bloodstream infections, responsible for 30–40% of these infections [4]. A study found a CoNS prevalence of 6–68% in suspected human infections in Africa within the last decade [2]. Of the CoNS, *Staphylococcus epidermidis* is the most common cause of human infection, culpable for about 24–80% of these infections [3]. 

CoNS are noted for their ability to develop antibiotic resistance against commonly used antibiotic classes such as β-lactams, aminoglycosides, and macrolides, with exceptionally high reported methicillin resistance rates [2] as well as resistance to antibiotics of last resort such as the glycopeptides [7]. Antibiotic resistance genes conferring resistance to these antibiotic classes can be transferred between staphylococcal species such as *S. aureus* and *S. intermedius,* limiting the therapeutic options available [1,8].

This study describes the incidence of MDR CoNS from three hospitals in the uMgungundlovu District in the KwaZulu-Natal Province in South Africa.

## 2. Results

### 2.1. Isolation, Identification, and Species’ Distribution

**Figure 1:**
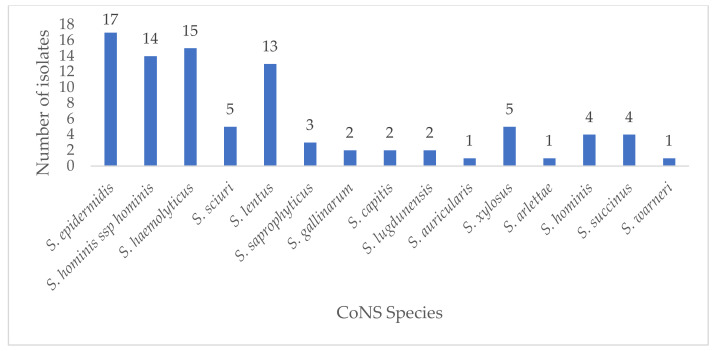
Distribution of CoNS isolates in this study.

Eighty-nine (89) out of the 130 presumptive isolates were confirmed as CoNS by the automated VITEK 2 system and used for further analysis. Of these, *Staphylococcus epidermidis* (17, 19.1%) was the most frequent species identified. Identities of other CoNS isolates were *S. hominis* ssp. *hominis* (14, 15.7%), *S. haemolyticus* (15, 16.9%), and *S. lentus* (13, 14.6%) (Figure 1). In terms of the distribution of CoNS by department, the emergency department (3), pediatric Outpatient Department (OPD) (3), and pediatric and extension wards (2) recorded the highest number of *S. epidermidis* isolates. *S. hominis* ssp. *hominis* had the highest distribution in the pediatric OPD (4) and medical ward (3). In the intensive care unit (ICU), *S. haemolyticus* (3) was the most frequently isolated, while *S. hominis* ssp. *hominis* (1), *S. lentus* (1), and *S. warneri* (1) were also found in the ICU. The rest of the species were sparingly distributed across all departments. Of the remaining 41 non-CoNS isolates, *Enterococcus faecalis* (12), *Enterococcus faecium* (8), *S. aureus* (6), and *Aerococcus viridans* (6) were the most abundant. Others were *Leuconostoc mesenteroides* ssp. *cremoris* (5), *Enterococcus columbae* (1), and *Dermacoccus nishinomiyaensis* (1).

### 2.2. Antibiotic Resistance Phenotypic Patterns of CoNS and mecA Detection

Of the 89 isolates, 68 (76.4%) were putatively methicillin-resistant by the phenotypic cefoxitin screen test. All isolates displayed resistance to at least one agent in one antibiotic class. High levels of antibiotic resistance rates were recorded for penicillin (100.0%), erythromycin (74.2%), azithromycin (74.2%), cefoxitin (76.4%), and trimethoprim/sulfamethoxazole (68.5%). Isolates were highly susceptible to linezolid (95.5%), gentamicin (95.5%), tigecycline (94.4%), nitrofurantoin (92.1%), amikacin (89.9%), vancomycin (86.5%), teicoplanin (82.0%), and ceftaroline (76.4%). The detailed phenotypic and genotypic profile of isolates is available in the supplementary Table S1. Table 1 delineates the percentage resistance of all CoNS, and methicillin-resistant coagulase-negative staphylococci (MRCoNS) isolates to the different antibiotics tested. None of the isolates was resistant to all antibiotics tested. Sixty-three (92.6%) of the 68 MRCoNS by cefoxitin phenotypic test were confirmed as MRCoNS by PCR detection of the *mec*A gene.

### 2.3. Multidrug Resistance and Multiple Antibiotic Resistance Index(MARI)

The MAR index ranged from 0.05 to 0.80, with an overall mean of 0.34 (Table 2 and Table 3). Multidrug resistance was observed in 68 (76.4%) of the isolates. Fifty-one antibiograms were observed (Table 4).

### 2.4. Demographic Characteristics of Patients and Statistical Analysis

**Figure 2:**
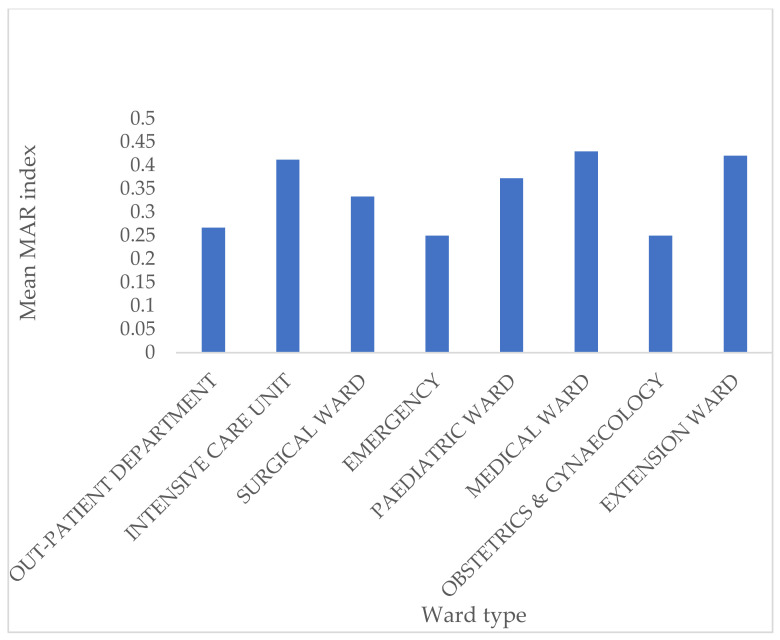
Distribution of MAR index against various ward types.

Patients’ ages ranged from 0 to 77. More than half (50.6%) of isolates were obtained from patients who were less than 1 year old. Of the remaining 49.4%, ages ranged from 1 to 77, with a mean age of 37.03 ± 23.22. Isolates were obtained from 46 (51.7%) males and 33 (37.1%) females, while seven (7.9%) were unknown/unspecified. Outpatients and inpatients made up 19.1% and 80.9% of samples, respectively. The distribution of isolates by wards was as follows: pediatric ward (17.9%), nursery (6.7%), emergency unit (5.7%), ICU (13.5%), medical ward (11.2%), surgical ward (6.7%), extension ward (7.9%), obstetrics and gynecology ward (1.1%), and maternity ward (1.1%). The Pearson Chi-Square test showed no significant association between the ward type and MAR index: *X*^2^(104, *n* = 89) =116.05, *p* = 0.197), even though the isolates with the highest MAR indices were from the ICU (Figure 2). Also, there was no statistically significant association between CoNS species and MAR index, *X*^2^(182, *n* = 89) = 203.07, *p* = 0.136). Furthermore, even though one-way ANOVA showed that *S. saprophyticus* isolates had a higher MAR mean (*p* = 0.012), the effect size was small (0.201). 

## 3. Discussion

This study describes the species distribution, antibiotic resistance profiles, MDR, and the MAR indices of clinical CoNS isolates from hospitals in the uMgungundlovu District of KwaZulu-Natal Province in South Africa. There was a diversity of CoNS species isolated, with *S. epidermidis* as the most abundant species, in agreement with previous studies conducted in ICUs investigating vancomycin heteroresistance and reduced glycopeptide susceptibility among bloodstream CoNS in Egypt and Italy [9,10]. Generally, the CoNS species distribution in this study agrees with detection rates of studies in Africa. A review paper [2] based on 35 studies in Africa, describing epidemiologically relevant CoNS found the most abundant CoNS species to be *S. epidermidis* and *S. haemolyticus* but found low detection rates of *S. capitis* (three studies), *S. lugdunensis* (three studies), *S. caprae* (one study), and *S. gallinarum* (one study). The MRCoNS prevalence rate (76.4%) was higher than that found in previous studies from clinical samples in healthcare settings in Nigeria (46.3%) [11] and Egypt (75.9%) [9]. However, higher MRCoNS prevalence figures of 86% [12] and 100% [13] were detected for CoNS implicated in infections in South Africa. The *S. epidermidis* group (*S. epidermidis* and *S. haemolyticus*) is an important cause of nosocomial infections [1]. *S. epidermidis* is the most frequently isolated staphylococcal species in humans and considered the most important CoNS species [3]. In this study, the *S. epidermidis* group was part of the three most abundant CoNS species. 

There was a 92.6% agreement between phenotypic and genotypic confirmation of methicillin resistance in this study. Methicillin resistance in isolates that lack the *mec*A gene may be mediated by other mechanisms of methicillin resistance, such as possession of *mec*C [14], *mec*B genes [15], or the overproduction of β-lactamases [2]. The development of methicillin resistance has been observed in about 80% of CoNS species, contributing to increased morbidity and mortality in hospitals due to their prominence in healthcare-associated infections (HAIs) [11]. 

The majority (76.4%) of the isolates in this study showed a multidrug resistance phenotype, with isolates displaying high resistance to commonly used antibiotics such as penicillin (100.0%), macrolides (74.2% each for erythromycin and azithromycin), and sulfamethoxazole/trimethoprim (68.5%). Similar high-resistance patterns have been observed against these antibiotics in other studies elsewhere [16,17]. However, the isolates in this study displayed high susceptibility against reserve antibiotics such as linezolid (95.5%) and the anti-MRSA (methicillin-resistant *Staphylococcus aureus*) β-lactam antibiotic ceftaroline (76.4%). Similarly, complete susceptibility of CoNS isolates was observed against vancomycin, tigecycline, teicoplanin, and linezolid in another South Africa study [13]. The high susceptibilities recorded against these antibiotics could be due to the reserved use of those antibiotics, mainly for resistant staphylococcal infections. Thus, the last resort antibiotics still retain high activity against CoNS and can be used for empirical treatment of conditions such as suspected CoNS sepsis, even though resistance against these antibiotics is gradually increasing [2]. The 51 antibiogram types seen in MDR isolates indicate a wide diversity of resistance phenotypes. Resistance to FOX, PEN, AZM, ERY, and SXT (R7) was the most frequently observed pattern in six (8.8%) MDR isolates. The next most common pattern was resistance to FOX, PEN, CIP, MXF, AZM, ERY, CLI, and RIF, observed in four (5.9%) MDR isolates. Due to its ability to penetrate biofilm, rifampicin is one of the preferred antibiotics for treating bone and joint infections [18]. However, the development of resistance due to continued use of the antibiotic has necessitated its use in combination with other antibiotics to treat bone and joint infections [18]. The resistance to rifampicin observed in this study (42.7%) means that the drug may not be relied upon alone in treating infections caused by CoNS.

Considering their susceptibility profiles, vancomycin, nitrofurantoin, linezolid, tigecycline, teicoplanin, gentamicin, amikacin, and the anti-MRSA cephalosporin ceftaroline retained high activities against CoNS in the study setting and may be relied upon in the treatment of CoNS infections. Decreasing vancomycin susceptibility is reported with increasing frequency in the clinically relevant CoNS literature and may be associated with increased vancomycin exposure [19]. The current study recorded 13.5% CoNS isolates with intermediate susceptibility to vancomycin. It is imperative to mitigate the development of vancomycin non-susceptibility, considering the vital role of glycopeptides in treating multidrug-resistant infections. 

Multidrug resistance (MDR) in CoNS is problematic in low/middle-income countries due to the limited access to newer antibiotics and the high cost of alternative treatment [2]. The study showed that most isolates were multidrug-resistant, with 71.9% of isolates having MAR indices of >0.20 and 18 (20.2%) had MAR values of ≥0.50. Other studies have, as well, recorded high MDR rates of CoNS [11]. MAR values higher than 0.2 indicate isolates, possibly originating from environments where antibiotics are frequently used, and may also hint at possible nosocomial transmission within the hospital setting [20]. There was no statistically significant association between the type of ward and the MAR index (*p* = 0.197), even though some isolates with high MAR indices were recovered in the neonatal ICU. 

It is important to note that more than half (50.6%) of the patients in this study were less than 1 year old. This is important considering that neonates have underdeveloped immune systems, making them prone to HAIs, especially in ICUs [2]. CoNS are also a leading cause of bacteremia in neonatal ICUs [2], and neonatal sepsis is quite common in neonates with poor perinatal events. Considering that CoNS are recognized neonatal pathogens in upper- and high-income countries, they should be given equal attention in low- and middle-income countries [21]. 

That the ICU had the highest number of recovered isolates is significant as CoNS are frequently isolated in bloodstream infections in ICU patients [2]. The use of invasive devices such as catheters, especially in ICUs, increases the risk of infection by CoNS, which is further facilitated by biofilm formation. The study was, however, limited by the lack of clinical data that precluded an analysis of risk factors associated with pathogenicity and/or contamination, warranting detailed epidemiological data.

## 4. Materials and Methods

### 4.1. Ethical Considerations 

The study was approved by the Biomedical Research Ethics Committee of the University of KwaZulu-Natal (Reference: BREC/00001302/2020). This study was a substudy of the overarching research program on Antibiotic Resistance and One Health (Reference: BCA444/16).

### 4.2. Study Setting, Sample Collection, and Identification 

A total of one hundred thirty (130) suspected staphylococcal isolates were recovered from routine clinical specimens processed by the central microbiology laboratory for the uMgungundlovu District between October 2019 and February 2020. Isolates were obtained from blood cultures from both outpatients and inpatients, the latter from the intensive care unit (ICU), neonatal intensive care unit (NICU), pediatric ward, pediatric outpatient department (OPD), emergency departments, surgical ward, and nursery. 

Presumptive identification was undertaken by Gram staining, colony morphology on blood agar, and the catalase test. The Staphaurex™ Latex Agglutination Test (Thermo Scientific, Kent, UK) was used to differentiate staphylococci based on their coagulase activity. Speciation was undertaken using the automated VITEK 2 system (BioMérieux, Marcy-L’Etoile, France). Patients’ demographic data (age, sex, ward type, and specimen source) were obtained from anonymous patient records. Isolates were stored at −86 °C in tryptic soy broth (Oxoid Ltd., Basingstoke, UK) containing 10% glycerol (VWR Lifescience Biotechnology, Missouri, TX, USA) and used for further analyses. 

### 4.3. Antimicrobial Susceptibility Testing and Determination of Methicillin-Resistant Coagulase-Negative Staphylococci (MRCoNS) 

Antibacterial susceptibility profile of isolates against a selected antibiotic panel was ascertained by the Kirby–Bauer disk diffusion method and interpreted according to the Clinical and Laboratory Standard Institute (CLSI) [22] guidelines using the following antibiotic discs: penicillin G (10 µg), cefoxitin (30 µg), ceftaroline (30 µg), ciprofloxacin (5 µg), moxifloxacin (5 µg), azithromycin (15 µg), erythromycin (15 µg), gentamicin (120 µg), amikacin (30 µg), chloramphenicol (30 µg), tetracycline (30 µg), doxycycline (30 µg), teicoplanin (30 µg), tigecycline (15 µg), linezolid (30 µg), clindamycin (10 µg), rifampicin (5 µg), sulfamethoxazole/trimethoprim (1.25/23.75 µg), and nitrofurantoin (300 µg). The cefoxitin test (disk diffusion) was used to screen methicillin resistance [22]. All disks were purchased from Oxoid (Oxoid Ltd., Basingstoke, UK). Multidrug resistance was defined as resistance to at least one agent in three or more distinct antibiotic drug classes. Susceptibility testing for vancomycin was done by minimum inhibitory concentration (MIC) (according to the CLSI guidelines) using the broth microdilution method due to the absence of breakpoints for the disc diffusion method [22]. *Staphylococcus aureus* ATCC 25923 and *Staphylococcus epidermidis* ATCC 12228 were used as the control strains. The multiple antibiotic resistance (MAR) index was calculated using the formula MAR = x/y, where x is the number of antibiotics an isolate displayed resistance toward and y is the total number of antibiotics tested against the isolate. The MAR index was used as an indicator of health risk assessment to identify if isolates originate from high or low antibiotic use environments.

### 4.4. DNA Extraction and PCR Amplification of MecA 

Genomic DNA was extracted using the GeneJET Genomic DNA Purification Kit (Thermo Fisher Scientific Baltics, Vilnius, Lithuania) according to the manufacturer’s instructions. The purity and concentration of extracted DNA were determined by Nanodrop™ 1000 spectrophotometer (Thermo Scientific, Wilmington, DE, USA) and stored at −20 °C for PCR. PCR detection of the *mec*A gene was done for presumptive methicillin-resistant coagulase-negative staphylococci (MRCoNS) isolates. The *mec*A gene, conferring resistance to methicillin, was amplified using the T100™ Thermal cycler (Bio-Rad, Hercules, CA, USA), using the primer set F-AACAGGTGAATTATTAGCACTTGTAAG and R-ATTGCTGTTAATATTTTTTGAGTTGAA (Inqaba Biotech, Pretoria, South Africa) [23], generating a 174 base pair fragment [24]. 

PCR was performed in a 25-µL reaction mixture containing 12.5 µL DreamTaq Green PCR Master Mix (ThermoScientific, Carthage, CA, USA), 0.5 µL each of forward and reverse primers, and 3 µL of template DNA. The PCR protocol was denaturation at 94 °C for 5 min, 35 cycles of 94 °C for 30 s, 55 °C for 45 s, and 72 °C for 45 s, and a final extension step of 10 min at 72 °C. PCR products were run on 1.5% agarose gel at 120 V for 60 min in a 1× Tris-acetate-EDTA (TAE) buffer (BioConcept Ltd., Basel, Switzerland) and visualized by UV transillumination using Bio-Rad ChemiDoc™ MP System (Bio-Rad, Hercules, CA, USA). *S. aureus* ATCC 43300 was used as a positive control.

### 4.5. Statistical Analysis 

Statistical analysis was carried out using Microsoft Excel 2016 (Microsoft Corperation, Redmond, DC, USA) and Statistical Package for the Social Sciences v. 26 (IBM Corperation, Armonk, NY, USA). Possible relationships between variables were investigated using the Pearson Chi-Square test and one-way analysis of variance (ANOVA). A *p*-value of <0.05 was considered statistically significant.

## 5. Conclusions

The study reports relatively high percentages of methicillin and multidrug resistance among CoNS isolates with a wide range of MAR indices. Considering that CoNS naturally inhabit the skin and mucous membranes, they may only be contaminants of clinical specimens. However, they are increasingly emerging pathogens, necessitating due diligence when recovered from clinical specimens.

## Figures and Tables

**Table 1:** Antimicrobial susceptibility pattern of CoNS and MRCoNS isolates from clinical sources.

Antibiotic	CoNS Isolates (*n* = 89)			MRCoNS Isolates (*n* = 68)		
	No of Susceptible (%)	No of Intermediate (%)	Number Resistant (%)	No of Susceptible (%)	No of Intermediate (%)	Number of Resistant (%)
Cefoxitin	21 (23.6)	NA	68 (76.4)	0 (0.0)	NA	68 (100.0)
Penicillin G	0 (0)	NA	89 (100)	0 (0.0)	NA	68 (100.0)
Ceftaroline	68 (76.4)	12 (13.5)	9 (10.1)	50 (73.5)	12 (17.6)	6 (8.8)
Ciprofloxacin	41 (46.1)	10 (11.2)	38 (42.7)	25 (36.8)	7 (10.3)	36 (52.9)
Moxifloxacin	46 (51.7)	6 (6.7)	37 (41.6)	30 (44.1)	5 (7.4)	33 (48.5)
Azithromycin	19 (21.3)	4 (4.5)	66 (74.2)	8 (11.8)	3 (4.4)	57 (83.8)
Erythromycin	17 (19.1)	6 (6.7)	66 (74.2)	6 (8.8)	5 (7.4)	57 (83.8)
Gentamicin	85 (95.5)	2 (2.2)	2 (2.2)	64 (94.1)	2 (2.9)	2 (2.9)
Amikacin	80 (89.9)	5 (5.6)	4 (4.5)	61 (89.7)	3 (4.4)	4 (5.9)
Chloramphenicol	64 (71.9)	2 (2.2)	23 (25.8)	47 (69.1)	2 (2.9)	19 (27.9)
Tetracycline	62 (69.7)	4 (4.5)	23 (25.8)	44 (64.7)	3 (4.4)	21 (30.9)
Doxycycline	65 (73.0)	3 (3.4)	21 (23.6)	46 (67.4)	2 (2.9)	20 (29.4)
Teicoplanin	73 (82.0)	10 (11.2)	6 (6.7)	59 (86.8)	4 (5.9)	5 (7.4)
Tigecycline	84 (94.4)	NA	5 (5.6)	64 (94.1)	NA	4 (5.9)
Linezolid	85 (95.5)	NA	4 (4.5)	65 (95.6)	NA	3 (4.4)
Clindamycin	47 (52.8)	10 (11.2)	32 (35.9)	32 (47.1)	7 (10.3)	29 (42.6)
Rifampicin	51 (57.3)	0 (0.0)	38 (42.7)	34 (50.0)	0 (0.0)	34 (50.0)
Sulfamethoxazole/trimethoprim	24 (26.9)	4 (4.5)	61 (68.5)	16 (23.5)	3 (4.4)	49 (72.1)
Nitrofurantoin	82 (92.1)	2 (2.2)	5 (5.6)	63 (92.6)	2 (2.9)	3 (4.4)
Vancomycin *	77 (86.5)	13 (13.5)	0 (0.0)	62 (91.2)	6 (8.8)	0 (0.0)

* Susceptibility against vancomycin was determined using the broth microdilution method.

**Table 2:** Multiple antibiotic resistance index (MARI) of CoNS isolates.

MAR Index	Number of Isolates
0.05	5 (5.6%)
0.10	5 (5.6%)
0.15	10 (11.2%)
0.20	5 (5.6%)
0.25	10 (11.2%)
0.30	6 (6.7%)
0.35	8 (8.9%)
0.40	12 (13.5%)
0.45	10 (11.2%)
0.50	8 (8.9%)
0.55	2 (2.2%)
0.60	6 (6.7%)
0.70	1 (1.1%)
0.80	1 (1.1%)

**Table 3:** Distribution of CoNS isolates depending on MARI value >0.2 in various departments.

Department	Number of Isolates with MAR Index > 0.2 (*n* = 64)	Percentage
Emergency	5	7.8%
ICU	11	17.2%
Medical Ward	10	15.6%
Obstetrics/gynecology	1	1.6%
OPD	7	10.9%
Surgical Ward	5	7.8%
Extension Ward	7	10.9%
Pediatric Ward	9	14.1%

**Table 4:** Resistance pattern observed in MDR CoNS (*n* = 68).

Resistance Pattern ^1^	Number
FOX-PEN-CIP-MXF-AZM-ERY-GEN-CLI-SXT	1
FOX-PEN-CIP-MXF-AZM-ERY-CHL-CLI-RIF-SXT	3
PEN- AZM-ERY-CHL-CLI-SXT	1
PEN-CPT-CHL-RIF-NIT	1
FOX-PEN-CIP-MXF-AZM-ERY-CLI-RIF	4
FOX-PEN-CIP-MXF-AZM-ERY-GEN-DOX-RIF-SXT	1
FOX-PEN-CIP-MXF-AZM-ERY-AMK-CLI-RIF	1
FOX-PEN-CIP-MXF-AZM-ERY-CHL-TET-DOX-TGC-TEC-LZD-CLI-RIF-SXT-NIT	1
FOX-PEN-MXF-AZM-ERY-CHL-CLI	1
PEN-AZM-ERY-SXT	3
FOX-PEN-AZM-ERY-SXT	6
PEN-CPT-MXF- CHL-TET-DOX-TGC-TEC-LZD-RIF-SXT-NIT	1
FOX-PEN-CIP-MXF-AZM-ERY-CLI-RIF-SXT	1
FOX-PEN-CIP-MXF-AZM-ERY-TET-DOX-RIF-SXT	2
FOX-PEN-AMK-CHL-TET-DOX-TGE-TGC-LZD-RIF-SXT-NIT	1
FOX-PEN-CPT-CIP-MXF-AZM-ERY-CHL-TET-DOX-CLI-SXT	1
FOX-PEN-CIP-MXF-AZM-ERY-CHL-TET-DOX-TGC-TEC-LZD-RIF-SXT-NIT	1
PEN- CIP-MXF-AZM-ERY-CHL-CLI-RIF-SXT	1
FOX-PEN-CIP-MXF-AZM-ERY-SXT	1
FOX-PEN-CIP-MXF-AZM-ERY-CHL-TET-DOX-CLI-RIF-SXT	2
FOX-PEN-MXF-AZM-ERY-SXT	1
FOX-PEN-CIP-MXF-AZM-ERY-AMK-CHL-CLI-SXT	1
FOX-PEN-CIP-MXF-AZM-ERY-TET-CLI-RIF	1
FOX-PEN-AZM-ERY-CLI-RIF-SXT	1
FOX-PEN-CPT-MXF-AZM-ERY-TET-DOX-CLI-RIF-SXT	1
FOX-PEN-CPT-CIP-MXF-AZM-ERY-AMK-CHL-CLI-RIF-SXT	1
FOX-PEN-CIP-MXF-AZM-RIF-SXT	1
FOX-PEN-AZM-ERY-CHL-TET-DOX-SXT	2
PEN-MXF-SXT	1
FOX-PEN-CIP-MXF-AZM-ERY-CLI-RIF-SXT	1
FOX-PEN-CIP-MXF-AZM-ERY-TET-DOX-CLI-RIF-SXT	1
FOX-PEN-AZM-ERY-TET-CLI	1
PEN-CIP-MXF-AZM-ERY-TET-CLI-RIF-SXT	1
FOX-PEN-CIP-AZM-ERY-TET-DOX-RIF-SXT	1
FOX-PEN-TET-DOX-TGC-RIF	1
FOX-PEN-AZM-ERY-RIF	1
FOX-PEN-CPT-AZM-ERY-TET-DOX-SXT	2
FOX-PEN-AZM-ERY-CHL-SXT	1
FOX-PEN-AZM-ERY-CHL-CLI-RIF-SXT	2
FOX-PEN-CIP-MXF-AZM-ERY-CLI-SXT	1
FOX-PEN-CIP-AZM-ERY-TET-DOX-SXT	1
FOX-PEN-CPT-CIP-AZM-ERY-CLI-RIF-SXT	1
FOX-PEN-CIP-AZM-ERY-TET-DOX	1
FOX-PEN-CIP-MXF-AZM-ERY-CHL-TET-DOX-SXT	1
FOX-PEN-CIP-AZM-ERY-CLI-SXT	1
FOX-PEN-CIP-MXF-ERY-RIF	1
FOX-PEN-AZM-ERY-TEC-RIF-SXT	1
FOX-PEN-AZM-ERY-TEC	1
FOX-PEN-CIP-MXF-AZM-ERY-CHL-CLI-SXT	1
FOX-PEN-CIP-MXF-AZM-ERY-RIF	1
FOX-PEN-CIP-MXF-RIF	1

^1^ FOX = cefoxitin; PEN = penicillin G; CPT = ceftaroline; CIP = ciprofloxacin; MXF = moxifloxacin; AZM = azithromycin; ERY = erythromycin; GEN = gentamicin; AMK = amikacin; CHL = chloramphenicol; TET = tetracycline; DOX = doxycycline; TEC = teicoplanin; TGC = tigecycline; LZD = linezolid; CLI = clindamycin; RIF = rifampicin; SXT = sulfamethoxazole/trimethoprim; NIT = nitrofurantoin.

## Data Availability

Not applicable.

## Supplementary Materials

The following are available online at https://www.mdpi.com/2079-6382/10/2/198/s1. Table S1: Detailed phenotypic and *mecA* profile of isolates.
