# Supplementary material for: Multidrug-Resistant Coagulase-Negative Staphylococci Isolated from Bloodstream in the uMgungundlovu District of KwaZulu-Natal Province in South Africa: Emerging Pathogens
Source: Antibiotics (Basel). 2021 Feb 18;10(2):198. doi: 10.3390/antibiotics10020198 (PMC7922184; doi:10.3390/antibiotics10020198)
Supplement: Supplementary file 1 [file antibiotics-10-00198-s001.pdf]

antibiotics

Article

Multidrug-Resistant Coagulase-Negative Staphylococci Isolated from Bloodstream in the uMgungundlovu District of KwaZulu-Natal Province in South Africa: Emerging Pathogens

Jonathan Asante [1,2,*], Bakoena A. Hetsa [1,2], Daniel G. Amoako [1,3], Akebe Luther King Abia [1], Linda A. Bester [4] and Sabiha Y. Essack [1]

[1] Antimicrobial Research Unit, College of Health Sciences, University of KwaZulu-Natal, Durban 4000, South Africa; hetszab@gmail.com (B.A.H.); amoakodg@gmail.com (D.G.A.); lutherkinga@yahoo.fr (A.L.K.A.); essacks@ukzn.ac.za (S.Y.E.)
[2] School of Laboratory Medicine and Medical Sciences, University of KwaZulu-Natal, Durban 4000, South Africa
[3] Centre for Respiratory Diseases and Meningitis, National Institute for Communicable Diseases, Johannesburg 2131, South Africa
[4] Biomedical Research Unit, University of KwaZulu-Natal, Durban 4000, South Africa; besterl@ukzn.ac.za
* Correspondence: josante33@yahoo.com

Table S1. Detailed phenotypic and *mecA* profile of isolates.

Isolate ID	CoNS Species	Ward Type	*MecA*	Antibiotic Resistance Profile																		
				FOX	PEN	CPT	CIP	MXF	AZM	ERY	GEN	AMK	CHL	TET	DOX	TEC	TGC	LZD	CLI	RIF	SXT	NIT
C1	*S. epidermidis*	Paediatric OPD	NA		R																R	
C3	*S. epidermidis*	Ward & nursery	-	R	R		R	R	R	R	R								R		R	
C4	*S. epidermidis*	A1 paediatric ward	-	R	R		R	R	R	R			R						R	R	R	
C5	*S. hominis ssp hominis*	Paediatric OPD	NA		R				R	R			R						R		R	
C6	*S. epidermidis*	Emergency department	NA		R	R							R							R		R
C7	*S. haemolyticus*	3N ICU	+	R	R		R	R	R	R									R	R		

C9	*S. epidermidis*	-	+	R	R		R	R	R	R	R				R					R	R		
C10	*S. haemolyticus*	ICU	+	R	R		R	R	R	R		R							R	R			
C11	*S. hominis ssp hominis*	Casualty	NA		R																R		
C13	*S. hominis ssp hominis*	Neonatal ICU	+	R	R		R	R	R	R			R	R	R	R	R	R	R	R	R		R
C17	*S. hominis ssp hominis*	H1 Medical ward	+	R	R			R	R	R			R						R				
C18	*S. haemolyticus*	KMMC Clinic	NA		R				R	R											R		
C19	*S. epidermidis*	Neonatal ICU	+	R	R				R	R											R		
C20	*S. hominis ssp hominis*	H2 Medical ward	NA		R	R		R					R	R	R	R	R	R		R	R		R
C21	*S. sciuri*	ICU	+	R	R		R	R	R	R									R	R	R		
C22	*S. lentus*	Ward D	+	R	R		R	R	R	R				R	R					R	R		
C23	*S. lentus*	Paediatric OPD	NA		R																		
C26	*S. lentus*	-	+	R	R																		
C27	*S. haemolyticus*	Ward D	+	R	R							R	R	R	R	R	R	R		R	R		R
C31	*S. haemolyticus*	A1 Paediatric ward	+	R	R				R	R													
C32	*S. sciuri*	E2 Paediatric ICU	+	R	R		R	R	R	R									R	R			
C35	*S. saprophyticus*	E1 Paediatric ward	+	R	R	R	R	R	R	R			R	R	R				R		R		
C36	*S. saprophyticus*	Neonatal ICU	+	R	R		R		R	R			R	R	R	R	R	R		R	R		R
C37	*S. lentus*	Neonatal ICU	NA		R		R	R	R	R			R						R	R	R		
C38	*S. epidermidis*	H2 Medical ward	+	R	R		R	R	R	R											R		
C39	*S. lentus*	5B2 Medical ward	+	R	R		R	R	R	R			R	R	R				R	R	R		
C40	*S. hominis ssp hominis*	3N Extension ward	+	R	R			R	R	R											R		
C42	*S. haemolyticus*	5F Medical ward	+	R	R		R	R	R	R				R	R					R	R		

C43	*S. lentus*	D1 Medical ward	+	R	R		R	R	R	R		R	R						R		R
C44	*S. gallinarum*	WARD E	+	R	R																
C47	*S. hominis ssp hominis*	D1 Medical ward	+	R	R				R	R											R
C48	*S. hominis ssp hominis*	3F Obstetrics & Gynaecol-ogy ward	+	R	R				R	R											R
C49	*S. capitis*	3N Extension ward	+	R	R		R	R	R	R				R					R	R	
C53	*S. lentus*	3N Main	-	R	R				R	R									R	R	R
C54	*S. haemolyti-cus*	3N Extension ward	+	R	R		R	R	R	R									R	R	
C55	*S. lentus*	Ward & Nursery	NA		R				R	R											
C56	*S. lentus*	E1 Paediatric ward	+	R	R	R		R	R	R				R	R				R	R	R
C57	*S. epider-midis*	7B2 Exten-sion ward	-	R	R	R	R	R	R	R		R	R						R	R	R
C58	*S. epider-midis*	Surgical OPD	+	R	R		R	R	R	R			R	R	R				R	R	R
C61	*S. haemolyti-cus*	D1 Medical ward	+	R	R		R	R	R											R	R
C66	*S. lentus*	Paediatric OPD	NA		R	R															R
C68	*S. epider-midis*	7F Paediatric ward	+	R	R				R	R			R	R	R						R
C72	*S. lentus*	2R Surgical ICU	NA		R																
C73	*S. lentus*	Ward F	NA		R																R
C74	*S. epider-midis*	Paediatric OPD	NA		R			R													R
C75	*S. lugdunen-sis*	-	NA		R																
C81	*S. hominis ssp hominis*	F2 Surgical ward	+	R	R		R	R	R	R									R	R	R
C87	*S. auricularis*	3N Main	+	R	R				R	R				R					R		
C93	*S. lentus*	H1 Medical ward	+	R	R		R	R	R	R			R						R	R	R

C100	*S. haemolyticus*	Emergency department	+	R	R				R	R							R
C102	*S. haemolyticus*	Paediatric OPD	+	R	R		R	R	R	R		R	R		R	R	R
C104	*S. capitis*	7F Paediatric ward	NA		R		R	R	R	R		R			R	R	R
C105	*S. hominis ssp hominis*	Paediatric OPD	NA	R	R		R		R	R		R	R			R	R
C107	*S. lugdunensis*	-	NA		R												
C110	*S. xylosus*	H2 Medical ward	+	R	R							R	R	R		R	
C113	*S. xylosus*	Medical OPD	+	R	R				R	R						R	
C114	*S. sciuri*	Medical OPD	NA		R				R	R							
C116	*S. hominis ssp hominis*	Paediatric OPD	NA		R				R	R							R
C118	*S. hominis ssp hominis*	Ward D	+	R	R	R			R	R		R	R				R
C119	*S. xylosus*	2F Paediatric ICU	+	R	R	R			R	R		R	R				R
C120	*S. gallinarum*	Paediatric OPD	+	R	R				R	R	R						R
C121	*S. hominis ssp hominis*	E1 Paediatric ward	+	R	R												R
C122	*S. hominis ssp hominis*	Paediatric OPD	+	R	R												R
C123	*S. haemolyticus*	Emergengy And Accident Unit	NA		R				R	R							
C125	*S. arlettae*	KMMC Clinic	+	R	R				R	R	R				R	R	R
C126	*S. epidermidis*	1F Male surgical Ward	NA		R												
C127	*S. hominis*	1F Male Surgical ward	+	R	R				R	R							R
C128	*S. hominis*	1F Male surgical Ward	+	R	R		R	R	R	R					R		R

C129	*S. haemolyticus*	1F Male Surgical ward	+	R	R		R	R	R	R					R	R	
C131	*S. sciuri*	Emergency and Accident Unit	+	R	R		R		R	R		R	R				R
C132	*S. sciuri*	C2 Surgical ward	+	R	R	R	R		R	R					R	R	R
C133	*S. epidermidis*	Paediatric OPD	+	R	R												R
C134	*S. epidermidis*	Casualty	+	R	R		R		R	R		R	R				
C135	*S. saprophyticus*	Paediatric OPD	+	R	R		R	R	R	R	R	R	R				R
C136	*S. succinus*	H Ward	NA		R				R	R							R
C137	*S. epidermidis*	Ward O	+	R	R		R		R	R					R		R
C138	*S. hominis*	H Ward	+	R	R												R
C139	*S. succinus*	3N Extension ward	+	R	R				R	R	R				R	R	R
C141	*S. haemolyticus*	3N Extension ward	+	R	R		R	R		R						R	
C142	*S. succinus*	Paediatric OPD	+	R	R												R
C143	*S. warneri*	3N ICU	+	R	R				R	R				R		R	R
C144	*S. epidermidis*	3N Extension ward	+	R	R		R	R	R	R	R				R	R	R
C145	*S. epidermidis*	Casualty	+	R	R				R	R				R			
C146	*S. haemolyticus*	Paediatric ward	+	R	R											R	R
C147	*S. xylosus*	Ward and Nursery	+	R	R		R	R	R	R	R				R		R
C148	*S. xylosus*	D5 Ward	+	R	R				R	R							
C149	*S. hominis*	KMMC Clinic	+	R	R				R	R							R
C150	*S. succinus*	Paediatric OPD	+	R	R				R	R	R	R	R				R
C151	*S. haemolyticus*	E1 Paediatric Ward	+	R	R		R	R	R	R						R	
C152	*S. haemolyticus*	ICU	+	R	R		R	R								R	

Abbreviations: FOX, cefoxitin; PEN, penicillin G; CPT, ceftaroline; CIP, ciprofloxacin; MXF, moxifloxacin; AZM, azithromycin; ERY, erythromycin; GEN, gentamicin; AMK, amikacin; CHL, chloramphenicol; TET, tetracycline; DOX, doxycycline; TEC, teicoplanin; TGC, tigecycline; LZD, linezolid; CLI, clindamycin; RIF, rifampicin; SXT, sulphamethoxazole/trimethoprim; NIT, nitrofurantoin; R, resistant; OPD, Outpatient Department; ICU, Intensive Care Unit; +, present; -, absent; NA: Not applicable.
